# Perspectives of Black Immigrant Women on Mental Health: The Role of Stigma

**DOI:** 10.1089/whr.2021.0071

**Published:** 2022-03-04

**Authors:** Aderonke Bamgbose Pederson, Elizabeth M. Waldron, J. Konadu Fokuo

**Affiliations:** ^1^ Department of Psychiatry and Behavioral Sciences, Feinberg School of Medicine, Northwestern University, Chicago, Illinois, USA.; ^2^ Department of Psychiatry, College of Medicine, University of Illinois, Chicago, Illinois, USA.

**Keywords:** black immigrant, mental health, stigma, spirituality, acculturation

## Abstract

**Background:**

Black immigrants are a major growing segment of the United States population. The intersection of race, gender, and migration places black immigrant women at the confluence of multiple social determinants of health, and thus, black immigrant women experience ongoing mental health disparities. Understanding their perspectives, mental health needs, and associated stigma is critical to promoting positive mental health outcomes.

**Methods:**

We conducted five focus groups (*N* = 22) among women from two black immigrant community organizations from February 2019 to June 2019. We used an inductive driven thematic analysis to identify codes and themes related to mental health and the role of stigma.

**Results:**

Overall five core themes associated with mental health and associated stigma concepts were found: The critical role of trusted sources and confidentiality, Conceptualization of mental illness and anticipated discrimination, Acculturative influence and migration as a source of emotional distress, Spirituality as a source of support and source of stigma, and Management of mental illness and addressing stigma.

**Conclusion:**

The conceptualization of mental illness and the associated stigma may be rooted in cultural and religious belief systems among black immigrants. Cultural beliefs and biopsychosocial models can coexist positively without interrupting the pathway toward optimized engagement in mental health care. Our mental health systems need to take these factors into consideration to implement programs that effectively serve black immigrant women's mental health needs.

## Introduction

There are 44 million immigrants and 23.3 million foreign-born immigrant women in the United States.^1^ The black immigrant population is a rapidly growing segment; yet, this population is largely understudied.^2–5^

The intersection of race, gender, and migration places black immigrant women at the confluence of multiple mental health disparities, which affect willingness to engage in mental health services, and warrants further research to address public health gaps.^6–8^ In a study describing results from the National Survey of American Life, Williams et al. described the importance of recognizing the considerable heterogeneity within the black population.^9^ Key areas of heterogeneity include ethnicity as well as immigrant history and status.^9^ This study showed that Caribbean men were more likely to be diagnosed with mood disorders compared to African American men,^9^ hence the mental health needs within the black population may vary across migrant status.

Past studies highlight barriers to engagement in mental health care for immigrants; however, few studies focus on black immigrants. It is well established that immigrants experience psychological stressors related to sociocultural adaptation to the host country.^10^ Acculturation (or stress related to migration), which includes difficulty assimilating to the beliefs, values, or social norms of the dominant culture, is a strong predictor of depressive symptoms and suicidal ideation among immigrants.^11^ Immigrants experience separation from their families, underemployment despite their education and linguistic barriers.^10,12^

Black immigrants underutilize mental health services despite the need for services.^13,14^ Black immigrants with mental health problems tend to present with physical symptoms (or somatization), which leads to missed and delayed diagnoses during routine primary care visits.^13,14^ One study showed that black immigrant women reported higher mental illness stigma concerns compared to U.S. born black and white women; however, questions on stigma were limited to three items using quantitative methods.^15^ Dedicated studies using qualitative methods highlighting the unique perspectives on mental health and the role of stigma as well as the mental health service needs of black immigrant women are needed.^14^

Studies have shown that stigma associated with mental illness is correlated with low use of mental health services despite need^13^ and is known to be a fundamental cause of health inequities for black immigrants.^8,16–18^ Stigma refers to a “social process involving labeling, stereotyping, separation, status loss, and discrimination in a power context”.^16^ There are several forms of stigma, including enacted stigma (behavioral manifestations of negative attitudes about people with a mental illness) and anticipated stigma (the expectation that one will be devalued for having mental illness and subjected to prejudice or discrimination).^19^

Black immigrants are more likely to seek mental health services through community resources such as traditional healers rather than health professionals due to factors related to stigmatizing beliefs.^20^ Past studies show mental illness stigma content of black immigrants includes beliefs that people with mental illness are weak, lazy, or morally flawed.^18^ Black immigrants may also have beliefs that mental illness is a punishment from God or caused by possession by evil spirits.^8,21,22^

Limited research on the specific conceptualization of mental health and the role of stigma among black immigrants hinders the effective development of interventions (such as antistigma programs) to promote engagement in mental health services.^4,23–25^ Underlying cultural beliefs such as the etiology of mental illness (*e.g.*, beliefs related to spiritual causes of mental illness compared to the biopsychosocial causes of mental illness) influences the willingness to seek mental health professional services.^26,27^ The aim of this study was to understand the perspectives of mental health and the role of stigma among black immigrant women given the limited studies focused on this population.

We seek to better describe the conceptualization of mental health and, therefore, inform the design and precision of mental health antistigma interventions for black immigrant women.

## Materials and Methods

### Ethical consideration

The Institutional Review Board at Northwestern University approved this study. All women were provided with and completed written and informed consent.

### Setting, participants, and study design

#### Setting

This study was conducted in Cook County, Illinois. This study was a result of a partnership between Northwestern University (U) and two black immigrant organizations [the Pan African Association and the United African Organization]. The U team included a psychiatrist and a research coordinator. Both community organizations provided support and programming to black immigrants from several different countries. An advisory board from both organizations was involved in planning the project.

#### Participants

The eligibility criteria for focus group participants (*N* = 22) were: (1) membership in two black immigrant organizations; (2) ages 18–65; (3) U.S. residency; (4) identify as female, and (5) English-language fluency. Convenience sampling was used. Each focus group comprised of 3–8 women. Demographic characteristics of participants are presented in Table 1.

**Table 1.** Participant Characteristics

	Overall (*n* = 22) *n* (%)
Age group	
18–24	8 (36.4)
25–40	7 (31.8)
41–65	6 (27.3)
Nonresponder	1 (4.5)
Marital status	
Married/partnered	9 (40.9)
Single/divorced	12 (54.5)
Nonresponder	1 (4.5)
Gender	
Female	22 (100)
Ethnicity^a^	
African	10 (45.5)
Afro-Caribbean	1 (4.5)
Black/African American	9 (40.9)
Other Hispanic	1 (4.5)
Nonresponder	1 (4.5)
Level of education	
High school/GED or less	7 (31.8)
Some college	8 (36.4)
Bachelor's degree or above	7 (31.8)
Source of income	
Family/friends	7 (31.8)
Government	1 (4.5)
Other	6 (27.3)
Self	8 (36.4)
Employment status	
No	10 (45.5)
Yes	11 (50.0)
Nonresponder	1 (4.5)
Income	
Less than $20,000	13 (59.1)
More than $20,000	6 (27.3)
Nonresponder	3 (13.6)
Are you a U.S. citizen?	
No	11 (50.0)
Yes	9 (40.9)
Nonresponder	2 (9.1)

^a^ Eligibility for the study was identifying as a black immigrant and a member of a black immigrant community organization.

GED, General Education Development.

#### Recruitment

Flyers and brochures were placed in local black immigrant community organizations. Community partners used text messages and word of mouth to advertise the study and recruit participants. The study contact information was distributed and interested community members were self-referred.

#### Interview procedure and protocol

The semistructured interview guide was developed by a multidisciplinary team, including community partners to ensure culturally acceptable language in discussing mental health, using “mind health” instead of the word “mental” in most cases. Community leaders recommended avoiding the use of the word “mental” in “mental health” because of negative connotations associated with the word “mental” on its own; this is consistent with studies showing that words can be a source of stigmatization in mental health.^28,29^ Rather than defining mind health, participants were asked what the phrase meant to them (focus group question 1).

We developed a focus group interview guide based on themes present in the existing literature and knowledge gaps around perspectives on mental illness.^7,15^ We completed five focus groups (*N* = 22) between February 23, 2019 and June 24, 2019. Each group lasted ∼60 minutes and was moderated by a member of the research team. All focus groups were held at the community organizations' conference room spaces.

Participants received $30 compensation for their time. All focus groups were audio recorded and recordings were transcribed. An experienced facilitator, who had previous experience conducting qualitative studies in a community setting (ABP) facilitated focus groups. In addition to the main facilitator, an assistant facilitator took notes to aid reliability and validity. It is important to note, that the main (ABP) and assistant facilitator are black immigrant women with lived experience as first- and second-generation immigrants, respectively.

Facilitators used probes to expand ideas and verify meaning expressed by participants. Throughout the interview, facilitators used member checking, rephrasing key responses to ensure accurate interpretation and increase validity and reliability of data.^30^ The assistant facilitator debriefed with the facilitator after the focus group session to ensure that accurate records were noted in real time.^31^ All participants' information was deidentified to ensure confidentiality.

### Data analysis

We were interested in the perspectives of mental illness, conceptualizations about emotional wellbeing, personal and community attitudes toward mental health, and content of stigmatizing views related to mental health among black immigrant women; the focus group questions are available in the Appendix Table A1.

We used a grounded theory approach to analyze the underlying meaning in the text. This includes an iterative process of coding, review, discussion, and revision.^32^ The goal of this form of thematic analysis is to capture predominant themes in relationship to a specific research area.^32,33^ NVivo software, a qualitative analysis tool, was used, which allows organization and analyzing of text-based data. Each focus group was audio recorded, then transcribed using Rev, a Northwestern University authenticated platform for transcription.

Transcripts were reviewed using five steps and entailed a continuous and iterative process.^32^ First, all responses were independently examined by both authors. Second, line by line coding to identify important meaning units was completed. Third, the meaning units were used to identify relevant codes, which were then grouped into higher order codes and themes. Fourth, coders met one to two times monthly (for 60 minutes duration) over 6 months to compare codes and themes, a code book was generated. Fifth, the coded transcripts were reviewed for consistency between the two coders. All inconsistencies were discussed and codes were revised until an intercoder reliability (*K* > 0.80) was reached. Coders applied the codebook to all transcripts.

## Results

Table 1 shows the demographic characteristics of participants. Eligibility for the study included identifying as black immigrants, we also asked about ethnicity in the demographic survey, some respondents (45%, *n* = 10) identified as African and some (40.9%, *n* = 9) identified as U.S. citizens and black American.

Majority of respondents were older than 24 years of age (59.1%, *n* = 15). Majority (59.1%, *n* = 13) identified as low income and half (50.0%) of the respondents reported having employment. One-third (31.8%) of respondents reported having a bachelor's degree or higher and one-third (31.8%) reported their main source of income was from family or friends. Majority of the sample reported (54.5%, *n* = 12) being single or divorced and some (40.9%, *n* = 9) reported being married or partnered (Table 1).

Five core themes related to mental health and mental health stigma emerged: (1) The critical role of trusted sources and confidentiality, (2) conceptualization of mental illness and anticipated discrimination, (3) acculturative causes of emotional distress, (4) spirituality as a source of support and sources of stigma, and (5) management of mental illness and addressing stigma. Appendix Table A2 shows a comprehensive list of participant quotes and highlighted themes. The themes were corroborated with existing knowledge about mental health stigma.^34–38^

### Theme 1: The critical role of trusted sources of support and confidentiality

The most prevalent theme among all respondents was the necessity for trust. This theme described how trust is promoted through shared identity (such as ethnicity) with health professionals and how mistrust reduces willingness to use health systems.

“So if a patient goes in a hospital for therapy, let's say I come from Congo, and someone else comes from Uganda, they should have someone who comes from that place. They would possibly understand them more. Not just telling someone who doesn't even know your background.” [FG3, Speaker 1]

“I will most likely talk to a friend, a family member about my mental illness or the things I'm going through, my depression or whatever I may have rather than talk to a doctor or anybody else clinical. Because I think that we have more of a connection and then we have that connection you build up a stronger support network.” [FG5, Speaker 4]

Several respondents described family as the main source of support. Some respondents believed community members were not trustworthy and might “stigmatize you.” Despite the consensus among several respondents that family was a main source of support and that shared backgrounds or identities enhanced trust, some respondents felt they preferred sharing with health professionals.

“When it comes to personal issues, when it comes to especially emotional issues, we really don't trust each other. Even some people appear to be trust worthy, but they stigmatize you, behind afterwards. So it's difficult for you to express your inner person, especially your emotional needs or your emotional health with people, even family members.” [FG5, Speaker 5]

### Theme 2: conceptualization of mental illness and anticipated discriminatory experiences

This theme described the conceptualization and attribution of underlying mental illness. This theme included biological theories, psychosocial frameworks, and community beliefs around insanity.

“I feel it's about body, your make up and your hormonal level. So hormones that deals with your mental status. If you have the low [levels], you are susceptible to having a breakdown faster than someone who has normal hormonal levels. So it is more medical.” [FG5, Speaker 8]

“It's biological because in Nigeria too that's why you don't marry, if someone, one person has gone mad in your family they believe that it's in your blood, so that means you can also go mad.” [FG4, Speaker 2]

Several respondents cited the fear of judgment as a reason that they would not share about their mental health or emotional distress and as the reason they would not seek mental health services. The stereotype of the “strong black woman” being able to self-manage mental illness was also highlighted.

“I grew up in the Bahamas part of the Caribbean and there's like a huge stigma on mental health. People look at you like you're crazy. In the Bahamas, growing up like you never heard of anyone reaching out if they had problems, mentally, because everyone just looked at you basically crazy.” [FG4, Speaker 4]

“So when I look at strength, when I look at how we handle the challenges you see the strength in every black woman, because we know how to conceal it, we know how to cover it and we know how to wear it and we have to find a solution to solving it.” [FG4, Speaker 3]

### Theme 3: Acculturation and Migration as a Source of Emotional Distress

Respondents described socioeconomic status loss with migration, the disparity between the expectation and reality of living in the United States, and feeling unwelcome. Respondents acknowledged the stress of acculturation. They also perceived that mental health services are focused on white people; therefore, these services did not cater to them. One respondent also describes their view of therapy saying: “even right now when I think of therapy or when I hear therapy, I think of a white person, to be honest.”

“You get depressed and stressed. Just like the way people come from Africa, different countries, thinking America is a wonderful happening paradise place. When you come here, what you face is another thing.” [FG1, Speaker 4]

“I would say in terms of stigmatization or the full understanding of emotional health issue has a lot to do with the cultural background. If we are talking about the western world people born and brought up here, it is easy for, for those individuals to come out, up front and say, “This is my problem.” But, when we look back at our cultural background, I'm talking about Africans, emotional well being has always had a stigma” [FG5, Speaker 2]

“The cultures also make it more difficult, being put in that box of cultural beliefs. Why shouldn't I be comfortable adjusting to the new changes in life, and that's one thing we have to educate the communities like, listen everybody is gonna go through changes from the moment you leave your home country to another country. You're gonna go through a phase of change, so these are changes that has to be addressed.” [FG4, Speaker 3]

### Theme 4: Spiritual Resources as Both a Source of Support and a Source of Stigma

Several respondents attributed mental illness to spiritual causes (such as possession by evil spirits), although some respondents explained that while religion is important, it may not explain all aspects of emotional distress. Some respondents described religious methods (such as prayer) as a means to address emotional distress. Other respondents described prayer as a tool that can support one's mental health in conjunction with therapy. One respondent described the benefit of having shared religious background with a therapist. Some respondents highlighted tensions that exist between seeking spiritual leadership as contrasted with using health resources like medications.

“When they see a mad person, ‘She's, he's cursed. He's evil.’ Or spiritual attack. Sometimes its life condition, what the person is going through. So it's both sides. The doctor's need to understand that there are things [that are] spiritual. And pastors need to understand, not everything [is] spiritual.” [FG1, Speaker 4]

“You have to call God. There is what we even call in Africa, deliverance [intense prayer]. If you're hearing voices just go to any church, any living churches around or mosque that deals with that, you'll be surprised within a week you're okay.” [FG4, Speaker 2]

“Because of faith, because of church, I guess. Yeah, which is true, like most pastors, they don't advise you to go medication.” [FG1, Speaker 4]

### Theme 5: Management of Mental Illness and Addressing Stigma

There was wide consensus that talking to other individuals or members of the community was a mechanism to improve mental health. The importance of professional support in addition to community support was highlighted. Some respondents minimized the seriousness of mental health and some reported the way to manage mental health was by talking to yourself. The use of physicians or medical care was seen as a last step for more severe cases of mental illness or suicidal thinking. Some respondents described the importance of educating themselves and the community as a means to reduce stigma. The use of contact-based interactions (or story telling by people with mental illness) was described positively.

“Maybe they can tell their stories and they could help out people they could tell them what they've been through and the coping mechanism they used, or how they went about it.” [FG2, Speaker 1]

“They were professionals, you know I was just like, ‘This is how the journey has been, let's go right back.’ And when they start peeling the pieces together you, you become a whole person in the process of allowing yourself to feel that [and] accept it, learn from it and grow from it. Because we're not strong enough to do this on our own.” [FG4, Speaker 3]

“You should be able to talk to yourself, your ability to talk to yourself, to calm yourself down will see you through difficult moments.” [FG5, Speaker 8]

## Discussion

The need for trust was a key theme and associated with other main themes, including discriminatory experiences, acculturative stressors, use of religious resources as a trusted space, and approach to managing mental illness. Some believed that sharing mental health concerns among family was safer than sharing with mainstream mental health professionals. Other participants felt that professional services offered higher levels of trust because of assumed confidentiality.^39^ The variations in trust mechanisms seemed to be less about the specific sources of support or care and more about maintaining shared values, including the expectation of privacy. Participants described that the building blocks of trust were connection, shared backgrounds (cultural competency), and confidentiality.

Existing studies highlight the importance of medical trust in the doctor patient relationship. Trust tends to vary by race with black people having lower trust levels in physicians compared to white people.^40^ In addition, our findings are consistent with the importance of confidentiality as a driving mechanism of trust.^40^ Our study extends the literature related to the black immigrant experience, which includes a need for providers who understand their unique lived experience as black immigrants, at times even more specific to their country of origin or specific ethnic identity.

Another topic present in several themes was the role of stress in mental illness and emotional distress. Sources of stress included finances, migration, and acculturation.^41^ Acculturation refers to the changes that occur as a result of contact with culturally dissimilar people or groups^42^ and acculturative stress is the physical and psychological stress that can result from this process.^43,44^ U.S.-based research in this area tends to focus on Hispanic/Latinx people, and black immigrants are understudied.^2^ Migration is considered one of the most significant sources of stress.^7^ For black immigrant women, the stress of migration is compounded by stress related to race in the American context and gender factors.^6^

Black women are at higher risk than other racial or gender groups to experience stress that may result in worse physical and mental illness, including higher rates of diabetes, heart disease, obesity, and depression.^6^ Our study highlights how specific factors related to the unique lived experience of black immigrant women contribute to negative health outcomes. Specific factors highlighted include the impact of immigration, lost opportunities with jobs, and feeling unwelcome in the United States. Some women also mentioned disappointment after witnessing gun violence and dangerous environments for their children, instead of the paradise they had envisioned of the United States.

The idea that therapy was for white people only was described as both a personal belief and a perceived societal belief. This raises the question of how our health systems may perpetuate the notion that mental health services are for a select few groups in society through implicit bias.^45^ Participants described past experiences (enacted stigma) and the anticipation of discrimination (anticipated stigma) in seeking support or counseling for mental health from a mental health professional. Discrimination against those with mental illness was described primarily from the experience of judgment within one's community.

In particular, the impact on family and community relationships was seen as a significant deterrent to disclosing mental health information to others. Some respondents saw secrecy as reflective of one's identity as a “strong black woman.” The cultural identity of the strong black woman represents both an image of resilience and a constricting archetype.^46,47^ To maintain the appearance of strength, black immigrant women may not seek necessary mental health support.

Religious values were central across themes. The role of spirituality is significant among black immigrants who tend to have their origins in African or Caribbean countries.^35–37,48^ Spiritual causes of mental illness included beliefs about curses, possession by spirits, or an attack from evil forces.^36,37^ In the focus group, spiritual practices such as prayer, support from religious leaders, and having enough faith were seen as mechanisms to manage mental illness.

The tension between accepted belief systems and health institutions was seen as a missed opportunity. Some respondents felt that their religious beliefs would be dismissed by health systems and this was a deterrent to seeking help. However, the experience of having health providers who were also religious or accepting of religious beliefs was seen as a strong facilitator toward seeking and engaging in mental health treatment.

Several respondents described beliefs around insanity or being crazy as a framework for understanding mental illness in the community. These beliefs, in most cases, were connected to beliefs formed in one's country of origin.^15,36^ Despite views on insanity, there was also broad acceptance of biopsychosocial models of mental illness, such as how hormones could result in mental illness. Some saw mental illness as curable while others saw mental illness as managed by will power. Moreover, the concept of contamination by association in marriage likely drives stigmatizing behaviors around social and physical distancing from people with mental illness.^36,49^

In addition, there was an appreciation of mental illness as a spectrum but this belief also led to some misconceptions that seeking care is only important when one is suicidal. Past research has demonstrated that a commonly held stigmatizing belief is that persons with mental illness are violent or dangerous.^36,50^ However, this belief was not prominent in this cohort of respondents.

Education and awareness forums to build mental health literacy and social contact have been described as ways to reduce stigma in our sample and in the larger literature.^4,23,51,52^ Storytelling is a widely accepted method of transmitting information and important in many African countries.^51,53^ It is crucial that stigma reducing interventions take into account the unique cultural belief systems among African immigrants.

There was wide consensus that reaching out to others was a means to manage negative feelings such as depression. While the importance of professional therapy was mentioned, it was not the main focus of seeking help. Talking about one's own mental illness, the underlying theory of social contact was a mechanism to reduce stigma and raise awareness in the community. Trust remained critical to vulnerability within the community.

**FIG. 1.**
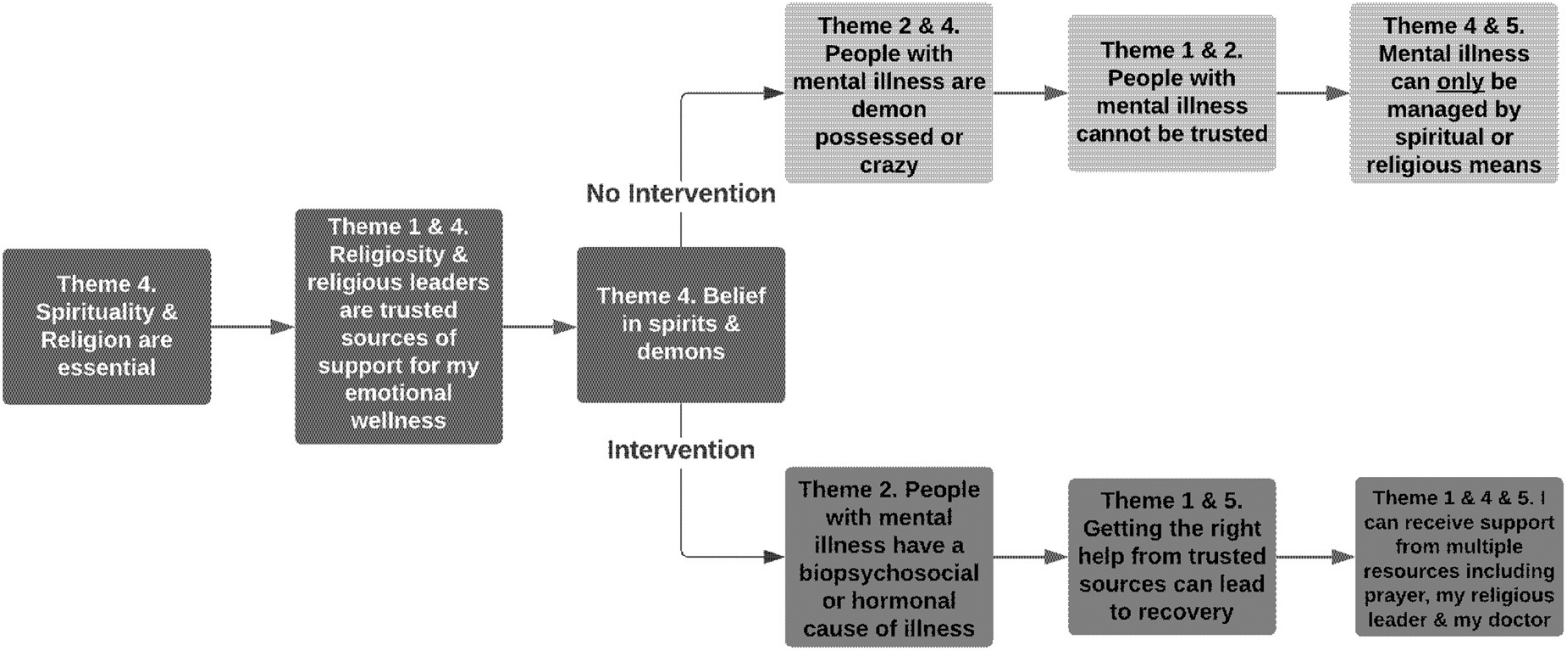
Theoretical flowchart based on core themes elicited and proposed intervention pathway.

We propose a model based on respondents' perspectives and emerging themes in this study on religiosity, use of religious leaders for support, beliefs in demon possession, and the resulting perspective on the use of mental health services. This pathway toward mental health services allows for the coexistence of religious- and culture-based beliefs along with the biopsychosocial model of disease. This is a theoretical model that we present for further exploration and research among black immigrant women, based on key themes in this study, toward optimizing mental health models that are culturally informed (Fig. 1).

### Practical implications

Mental health systems would benefit from increased diversity in the work force, including providers who identify as black immigrants. Family and community stakeholders are essential partners for mental health systems looking to build trust and improve mental health outcomes for black people with immigrant backgrounds. Cultural frameworks of mental illness may coexist positively with biopsychosocial frameworks. Dismissing cultural beliefs creates distance between black immigrants and mental health systems.

### Study limitations

While we present on several themes, they are not representative of the entire population of black immigrants living in the United States. We addressed this limitation by continuing focus groups until saturation was achieved. Nevertheless, future research may benefit from studies on perspectives on mental health among black immigrant women living in different geographic regions of the United States. Eligibility for the study was based on membership within a black immigrant organization; however, we did not explicitly assess generational status of respondents.

Future studies would benefit from understanding the perspectives of black immigrants based on ethnicity and generational status or length of stay in the United States. Future studies using mixed-method or quantitative methods of data collection examining the relationship between perspectives on mental health and use of mental health services would advance this line of research. We only conducted focus groups; however, key informant interviews afford more privacy. We addressed this limitation by having small focus group sizes and emphasized the importance of confidentiality within the groups.

## Conclusion

While stigmatizing views may be rooted in cultural beliefs, it is not the cultural beliefs themselves that should be equated with stigma. Rather, the interpretations of cultural beliefs may lead to stigmatizing views. Black immigrants are less trusting of mental health systems that reject the cultural beliefs that help anchor one within their community. Targeting the mechanisms that drive stigma using interventions such as social contact and education is likely to be more effective and culturally informed than attempting to alter one's underlying cultural beliefs.

## Abbreviation Used

GED: General Education Development

## Appendix

**Appendix Table A1.** Focus Group Questions

Focus group questions
1) What is mind health?
2) What are your perceptions of your community's understanding of stigma of mind health? Do you believe mind health is looked down upon in our society/community? In what ways?
3) Tell us about personal stigma of mind health: What are your personal beliefs about mind health, beliefs about mind health treatment and beliefs about seeking mind health treatment?
4) Stigma in different settings, at work, home or public community settings. Do people experience shame about mental illness at home or at work or in public settings? What can reduce the negative attitudes or feelings of shame?
5) Conceptualization of mental illness within the context and outside of the context of the biological human body: What is your understanding of the basis for depression, stress, anxiety or other experiences like these? Is there a biological explanation for these feelings or cultural or religious explanation? What are they? Which are more valid to you?
6) What strategies may be able to change and reduce stigma about difficult feelings like depression, stress, anxiety, or similar experiences and feelings?
7) Would you participate in these strategies? What are some barriers to participating?

**Appendix Table A2.** Core Themes and Representative Quotes

Identified themes	Representative quotes
Theme 1. The Critical Role of Trusted Sources of Support and Confidentiality “Everything we discuss is, I mean it's just between us”	“I will most likely talk to a friend, a family member about my mental illness or the things I'm going through, my depression or whatever I may have rather than talk to a doctor or anybody else clinical. Because I think that we have more of a connection and then we have that connection you build up a stronger support network.” [FG5, Speaker 4] “When it comes to personal issues, when it comes to especially emotional issues, we really don't trust each other. Even some people appear to be trust worthy, but yet they stigmatize you, behind afterwards. So it's difficult for you to express your inner person, especially your emotional needs or your emotional health with people, even family members.” [FG5, Speaker 5] “He is in a better position not to stigmatize me because he told me from the beginning of the conversation that, ‘Everything we discuss is, I mean it's just between us and all that.’ He's not gonna tell no one I know. So I felt free talking about my situation to a counselor rather than talking to someone in the community that probably would stigmatize me or judge me based on whatever is happening to me.” [FG5, Speaker 5] “So if a patient goes in a hospital for therapy, um, let's say I come from Congo, and someone else comes from Uganda, they should have someone who comes from that place. They would possibly understand them more. Not just telling someone who doesn't even know your background, who haven't even faced it, but if you tell somebody who has been through the same thing, they would understand more.” [FG3, Speaker 1]
Theme 2. Conceptualization of Mental Illness and Anticipated Discriminatory Experiences “People look at you like you're crazy”	“I feel it's about body, your make up and your hormonal level. So hormones that deals with your mental status. If you have the low (levels), you are susceptible to having a breakdown faster than someone who has normal hormonal levels. So it is more medical.” [FG5, Speaker 8] “Emotional health is the resiliency you have in your emotions. If a person were to go through something in their life, say like a death or whatever, of course mourning, grieving would be expected, it's even healthy.” [FG5, Speaker 4] “It's biological because in Nigeria too that's why you don't marry, if someone, one person has gone mad in your family they believe that it's in your blood, so that means you can also go mad.” [FG4, Speaker 2] “I grew up in the Bahamas part of the Caribbean and there's like a huge stigma on mental health. People look at you like you're crazy. In the Bahamas, growing up like you never heard of anyone reaching out if they had problems, mentally, because everyone just looked at you basically crazy.” [FG4, Speaker 4] “So if you're a person who is depressed, then that's the reason why people with depression, or stress, or anxiety don't voice it that much, because they know if I voice it and make it aware to everybody, there are positions that I'm not going to get. You might do something that is very normal, but because they know that you have the situation, that you cannot get some things correct, or accurate. You can do it, you cannot do some things as accurate as a person who is not under this condition.” [FG3, Speaker 1] “So when I look at strength, when I look at how we handle the challenges you see the strength in every black woman, because we know how to conceal it, we know how to cover it and we know how to wear it and we have to find a solution to solving it.” [FG4, Speaker 3]
Theme 3. Acculturative Influence and Migration as a Source of Emotional Distress “If we are talking about … people born and brought up here, it is easy for those individuals to come out”	“You get depressed and stressed. Just like the way people come from Africa, different countries, thinking America is a wonderful happening paradise place. When you come here, what you face is another thing.” [FG1, Speaker 4] “The cultures also make it more difficult, being put in that box of cultural beliefs. Why shouldn't I be comfortable adjusting to the new changes in life, and that's one thing we have to educate the communities like, listen everybody is gonna to go through changes from the moment you leave your home country to another country. It's a new chapter, it's a new beginning. You're gonna go through a phase of change, so these are changes that has to be addressed for people to even get it.” [FG4, Speaker 3] “we need counselors as we are coming in from the airport, if you can go to put it at the airport as we're coming in please come over here.” [FG4, Speaker 2] “In Africa we play outside, here they're stuck in the room. But right here it's only take festival for the kids to come out and stay together or you play in a park, you're scared of gunshots or somebody kidnapping your child or somebody raping or whatever.” [FG4, Speaker 5] “I would say in terms of stigmatization or the full understanding of emotional health issue has a lot to do with the cultural background. If we are talking about the western world people born and brought up here, it is easy for, for those individuals to come out, up front and say, ‘This is my problem.’ But, when we look back at our cultural background, I'm talking about Africans, emotional well being has always had a stigma” [FG5, Speaker 2]
Theme 4. Spirituality as both a Source of Support and a Source of Stigma: “If you're hearing voices just go to any church”	“To emphasize on, when they see a mad person, ‘She's, he's cursed. He's evil.’ Or spiritual attack. Sometimes its life condition, what the person is going through. And the doctors also need to understand … So it's both side. The doctors medication need to understand that there are things spiritually. And pastors, we need to understand, it's not everything that it's spiritually. Yes, everything is spiritually according to faith, but it's not everything that it's cursed or evil, or something.” [FG1, Speaker 4] “The spirits, they're attacking her, which is yes, we know because we are Christian, the Bible teaches or African, it contributes. But it's not always, we have to look and know that sometimes we get stressed because of our own personal things. So I think churches also can try at times when they do like self-meeting, apart from church services, tell the people apart from being too much spiritual on the point of stress, just to make people know that there's evil.” [FG1, Speaker 4] “You can pray for the person, but not saying that they're possessed. You can pray for the person like with mental illness like you could pray for a person who has a heart condition, but then still take them to the hospital.” [FG3, Speaker 1] “You have to call God. There is what we even call in Africa, deliverance. If you're hearing voices just go to any church, any living churches around or mosque that deals with that, you'll be surprised within a week you're okay.” [FG4, Speaker 2] “I wasn't expecting anything out of it. I mean this one will make me calm down through that situation and he (counselor) worked because he was also a spiritual person. In fact he asked me if I was spiritual, I said, ‘Yes.’ He said, ‘You can always talk to God about this.’” [FG5, Speaker 5] “Because of faith, because of church, I guess. Yeah, which is true, like most pastors, they don't advise you to go medication.” [FG1, Speaker 4]
Theme 5. Management of Mental Illness and Addressing Stigma: “talk to yourself.”	“My feelings on it is that I feel like you should reach out. And even me myself like I feel, a lot of personal things I'm going through I probably do need to reach out.” [FG4, Speaker 4] “They were professionals, you know I was just like, ‘This is how the journey has been, let's go right back.’ And when they start peeling the pieces together you, you become a whole person in the, in the journey of pouring it out, in the process of allowing yourself to feel that accept it, learn from it and grow from it. Because we're not strong enough to do this on our own, this life was not meant for us to do it by ourselves.” [FG4, Speaker 3] “You should be able to talk to yourself, your ability to talk to yourself, to calm yourself down will see you through difficult moments.” [FG5, Speaker 8] “So I think that instead of looking down on the person you can, if it's a person you can talk to, you can even talk to them or seek help from the doctor, because it's something that can be cured and taken care of.” [FG3, Speaker 1] “If someone is very suicidal, if they don't get the right treatment, they're going to go on with their mission and commit suicide. However, if they get the right help, if they're around the right people, if they're getting assisted, if they're taken serious. Then maybe there's hope for them.” [FG2, Speaker 2] “I think it is how people who are not emotionally ill negatively treat or think about people who are mentally ill. And that depends on what information they have or how much information they have about mental illness. So I think my final statement would be the higher the education, or the more the information, the lower the stigma.” [FG3, Speaker 1] “Maybe they can tell their stories and they could help out people they could tell them what they've been through and the coping mechanism they used, or how they went about it.” [FG2, Speaker 1]
